# Global evidence on the cost-effectiveness of cardiac resynchronization therapy for heart failure: a systematic review

**DOI:** 10.3389/fcvm.2026.1766979

**Published:** 2026-05-21

**Authors:** Kaltrina Bajraktari, Zanfina Ademi, Artan Bajraktari, Roberta Bajrami, Besfort Kryeziu, Robert J. Gil, Michael Y. Henein, Gani Bajraktari

**Affiliations:** 1 Faculty of Economy, AAB College, Prishtina, Kosovo; 2 Health Economics and Policy Evaluation Research, Faculty of Pharmacy and Pharmaceutical Sciences, Monash University, Melbourne, VIC, Australia; 3 Medical Faculty, University of Prishtina, Prishtina, Kosovo; 4 Clinic of Cardiology, University Clinical Centre of Kosova, Prishtina, Kosovo; 5 Department of Public Health and Clinical Medicine, Umeå University, Umeå, Sweden; 6 Department of Cardiology, National Medical Institute of the Ministry of Interior and Administration, Warszawa, Poland; 7 National Heart and Lung Institute, Imperial College London, London, United Kingdom; 8 Department of Medical Biotechnologies, Siena University, Siena, Italy; 9 Department of Life and Health Sciences, University of Nicosia, Nicosia, Cyprus

**Keywords:** cardiac resynchronization therapy, cost-Effectiveness, CRT-D, CRT-P, health economics, heart failure, ICER, QALY

## Abstract

**Background and aim:**

Heart failure (HF) remains a global public health challenge, with high morbidity, mortality, and healthcare costs despite advances in pharmacological and interventional therapies. Cardiac resynchronization therapy (CRT) reduces symptoms, hospitalizations, and mortality, but is associated with increased healthcare costs. This review aims to evaluate the existing evidence on the cost-effectiveness of CRT compared with standard optimal medical therapy.

**Methods:**

A systematic search of Web of Science, PubMed, and Scopus was conducted from January 2004 up to July 2025 to identify studies reporting economic outcomes such as quality-adjusted life years (QALYs) and incremental cost-effectiveness ratios (ICERs) for CRT treatment in addition to optimal medical therapy (OMT) compared to OMT only. The study was registered in PROSPERO. We included studies comparing CRT plus OMT with OMT alone, as well as CRT-P vs. CRT-D. Costs were converted to 2024 euros (€) and assessed against country-specific willingness-to-pay thresholds.

**Results:**

Eighteen studies met the inclusion criteria and were included in the final review CRT + OMT proved to be highly cost-effective across multiple healthcare settings: ICERs ranged from €3,048-€71,447/QALY gained. CRT-D compared with CRT-P showed more cost variability, with ICERs varying between €24,909-€105,572/QALY gained, often exceeding accepted country-specific willingness-to-pay thresholds.

**Conclusions:**

This review confirms that CRT, particularly CRT-P, is a cost-effective treatment for patients with HFrEF who remain symptomatic despite OMT. CRT-D had variable cost-effectiveness, thus should be reserved for selected high-risk patients. These findings support prioritizing CRT-P as a high-value therapy within advanced heart failure care.

**Systematic Review Registration:**

https://www.crd.york.ac.uk/prospero/display_record.php?ID=CRD420251171292, PROSPERO CRD420251171292.

## Graphical Abstract

**Figure:**
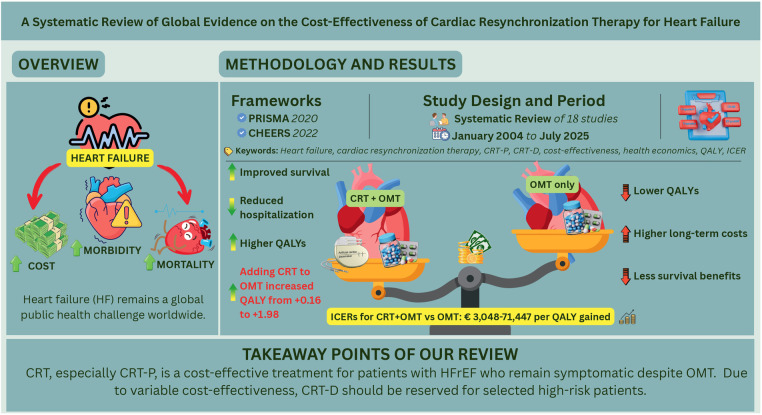
Summary of the systematic review evaluating the cost-effectiveness of cardiac resynchronization therapy (CRT) in heart failure. CRT combined with optimal medical therapy improves survival, increases quality-adjusted life years (QALYs), and is generally cost-effective, while CRT-D shows variable cost-effectiveness and should be reserved for selected high-risk patients.

## Introduction

The syndrome of heart failure (HF) remains a major public health challenge worldwide. The mortality and need for hospitalization of patients with HF remain very high, despite advances in diagnosis and treatment, particularly in the last decades (1). The introduction of new drugs (2) and new assist devices (3) resulted in significant improvement of clinical outcomes in HF patients, in particular those with HF and reduced left ventricular ejection fraction (LV EF) (HFrEF). In addition to treatment challenges, the cost of such advances has increased significantly (4). A significantly growing number of patients, especially those in aging populations, require frequent hospitalizations with its well-recognized cost implications on healthcare systems (3, 5). In the United States of America, the total cost of HF care was estimated at $43.6 billion in 2020, with projections reaching $69.7 billion by 2030, if healthcare strategies remain unchanged (6). Cardiac resynchronization therapy (CRT) has been introduced as an effective treatment for patients with HFrEF and LV desynchrony, mainly based on QRS duration measurements (7). Data from the major randomized clinical trials (RCT) such as CARE-HF (8), COMPANION (9), and RAFT (10), showed that in addition to patients' symptoms reduction and improved survival, CRT significantly reduced HF related hospitalizations, with consequent reduction in long-term healthcare costs (10), despite being, itself, an expensive procedure (11). Several studies have been conducted to assess the cost-effectiveness of CRT implantation, ensuring that it is both medically and financially beneficial (12). Despite this, the existing literature lacks a comprehensive synthesis that integrates clinical effectiveness and economic evidence to determine CRT cost-effectiveness (13). The objective of this systematic review, therefore, was to provide a detailed up-to-date analysis of the existing evidence on the cost-effectiveness of CRT for the treatment of HF patients, in different countries.

## Methods

This review was conducted in accordance with PRISMA (Preferred Reporting Items for Systematic Reviews and Meta-Analyses), and reporting quality was assessed using CHEERS (Consolidated Health Economic Evaluation Reporting Standards, 2022). The study protocol was registered in PROSPERO (CRD420251171292). The completed PRISMA 2020 and CHEERS 2022 checklists are provided in the Supplementary Material (Supplementary Tables S1, S2, respectively).

### Inclusion and exclusion criteria

The target population in the included studies consisted of adult patients aged ≥18 years with symptomatic heart failure (mostly NYHA class II–IV) and reduced LV ejection fraction (≤35%), as defined in each trial. The included studies had to be original research articles that: (1) Conducted an economic evaluation and reported quantitative cost-effectiveness outcomes, specifically quality-adjusted life years (QALYs) and incremental cost-effectiveness ratios (ICERs), (2) Compared CRT + OMT with OMT as only treatment, or compared CRT-Pacemaker (CRT-P) with CRT-Defibrillator (CRT-D), and (3) Were published in English. The exclusion criteria were studies with only partial economic evaluation, studies published as abstracts only, case reports and conference papers.

#### Search strategy

Two independent reviewers (K.B and A.B) developed and performed the literature search strategy using predefined Medical Subject Headings (MeSH). The search strategy covered the period from January 2004 to July 2025, as 2004 marks the beginning of the modern CRT era, following the pivotal COMPANION (2004), that established CRT as standard therapy in symptomatic patients despite OMT. A literature search was performed using three reliable databases: Web of Science, PubMed, and Scopus. The PubMed search strategy was structured as follows: (“cardiac resynchronization therapy” OR CRT OR “biventricular pacing” OR biventricular OR resynchronization OR “CRT-P” OR “CRT-D”) AND (“cost-effectiveness” OR “cost effectiveness” OR “cost-utility” OR “cost utility” OR “economic evaluation” OR economic* OR cost* OR ICER OR QALY) AND (“heart failure” OR “cardiac failure” OR HF) AND [English (Language)]. The syntax was adapted appropriately for Scopus and Web of Science. Searches were limited to studies published between January 2004 and July 2025 and to articles in English. Any discrepancies in search interpretation were resolved by consensus or by consulting a third reviewer (G.B). Disagreements about study inclusion were resolved by discussion, if consensus could not be reached, a third reviewer adjusted.

##### Research question and PICOS framework

**Figure 1:**
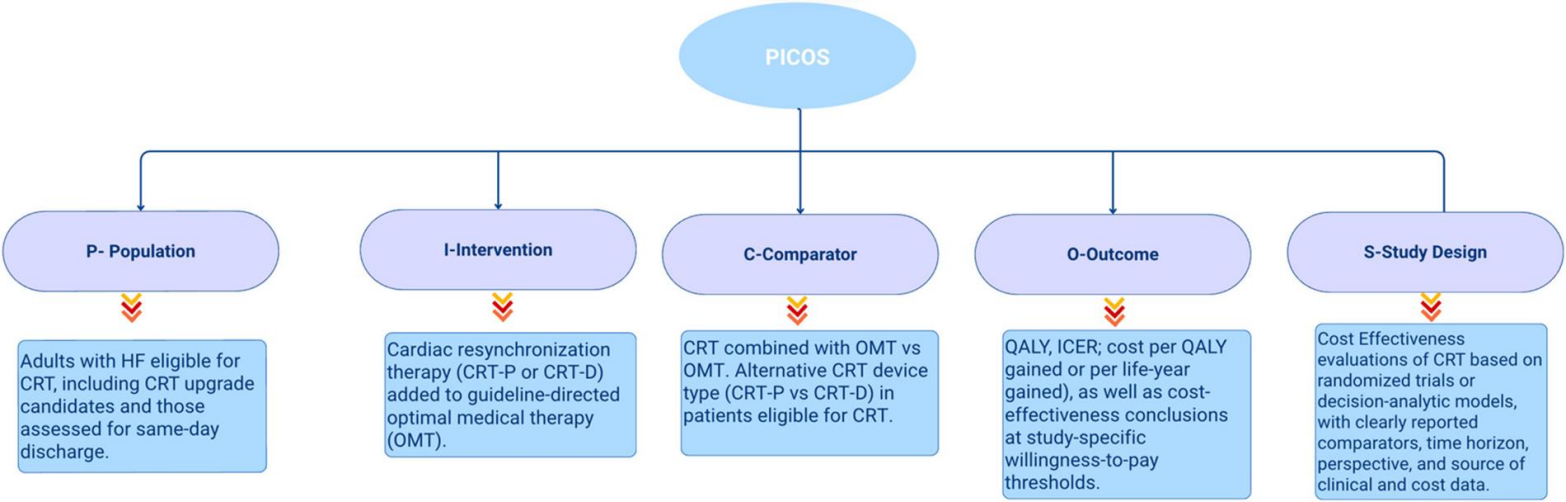
Figure created by the author based on the PICOS framework as described in Amir-Behghadami & Janati (2020).

The research questions guiding this systematic review were developed using the PICOS criteria (Population, Intervention, Comparator, Outcome, Study design) framework to ensure clarity, focus, and relevance in identifying and selecting studies for inclusion (Figure 1) (14). The research questions were:

- RQ1: *What is the cost-effectiveness of CRT when combined with OMT compared to OMT alone?*
- RQ2: *How does CRT-D compare to CRT-P in terms of incremental cost-effectiveness?*

#### Data extraction and synthesis

EndNote software (version 20, Clarivate Analytics) was used to organize the studies, review titles and abstracts, and identify duplicate articles. The final included studies in this systematic review were original research papers that reported cost-effectiveness outcomes for CRT. The PICOS questions were designed to guide data extraction, synthesis, and interpretation. From each study, the following data were extracted using a standardized form: author(s), publication year, country/region, study objective, model type (trial-based or Markov), perspective of the study, time horizon, discount rate and currency year, types of costs included, total costs, total effects (life-years and quality adjusted life years), ICERs, and results of sensitivity analyses. For analytical clarity, the included studies were categorized into two main comparison groups: (1) CRT or CRT-P vs. OMT; and (2) CRT-D vs. CRT-P. Each comparison was analyzed separately, and results were summarized in distinct tables (Tables 1, 2).

**Table 1:** Comparative summary of CRT-D or CRT-P vs. OMT across included studies.

Author, Year	Country	Population	Time Horizon	Follow Up	QALY gained	CRT-D/CRT-P	ICER (CRT)	ICER (€ 2,024, 3% adj.)	WTP thresholds	Perspective	Model type
Feldman et al., (37)	US	NYHA Class III–IV, LVE ≤ F35%, QRS ≥ 120 ms	7 years	2 years	+0.71	CRT-P	$19,600/QALY	€32,914/QALY	77,770€—233,310€	Healthcare payer	Markov simulation model.
							Cost-effective				
Banz et al. (22)	Germany	HF patients (NYHA III–IV, EF ≤ 35%)	5 years	5 years	+0.16	CRT-P	€36,600/QALY	€66,099/QALY	51,600€–154,800€	Healthcare payer	Comprehensive decision-analytic model with Markov sub models.
							Cost-effective				
					+0.84	CRT-D	$ 43,000/QALY	€71,447/QALY	51,600€–154,800€		
							Marginally cost-effective				
Calvert et al., (32)	UK	NYHA Class III–IV, LVEF ≤ 35%, QRS > 120 ms	Lifetime	29.4 months	+1.03	CRT-P	€19,319/QALY	34,880€/QALY	48,990–146,970€	Healthcare payer	Trial-based cost-utility analysis with extrapolation.
							Cost-effective				
Caro et al. (20)	UK	HF patients, NYHA Class III–IV	7 years	29.4 months	+0.43	CRT-P	£15,247/QALY gained; Cost-effective	€32,440/QALY	48,990€–146,970€	Healthcare payer	Discrete event simulation.
Yao et al., (35)	UK	HF patients, NYHA III/IV, LVEF ≤ 35%	Lifetime	2.5 years	+1.98	CRT-P	€7,538/QALY	€12,732/QALY	48,990€–146,970€	Healthcare payer	Markov model with Monte Carlo simulation.
							Cost-effective				
Blomström et al., (21)	Danmark	NYHA III–IV, LVEF < 35%, QRS > 120 ms (CARE-HF patients)	Lifetime	29.4 months	+0.91	CRT-P	€4,759/QALY (Denmark)	€7,636/ QALY Denmark	`65,340€–196,020€ (Denmark)	Healthcare payer	Trial-based cost–utility analysis with lifetime extrapolation.
	Finland										
							€3,571/QALY (Finland)	€5,728/QALY (Finland)			
	Sweden										
									48,900€–146,700 € (Finland)		
							€6,493/QALY (Sweden)	€10,420/QALY (Sweden)			
							Cost-effective				
									52,550€–157,650€ (Sweden)		
Callejo et al. (29)	Spain	HF patients (NYHA III–IV, EF ↓, QRS ↑)	Lifetime	18 months	+0.69	CRT-P	€28,612/QALY	€43,243/QALY	32,500€–97,500€	Public healthcare system	Markov decision-analytic model.
							Cost-effective				
Neyt et al., (34)	Belgium	NYHA III/IV, LVEF ≤ 35%	Lifetime	15.6 months	+1.31	CRT-P	€11,200/QALY	€16,434/QALY	30,000€–50,000€	Healthcare payer	Markov model.
							Cost-effective				
Maniadakis et al. (36)	Greece	NYHA III–IV, EF ≤ 35%, QRS > 120 ms	Lifetime	29.4 months	+1.41	CRT-P	€6,045/QALY	€8,872/QALY	22,650€–67,950€	Healthcare payer	Trial-based cost-utility analysis with lifetime extrapolation.
							Cost-effective				
Poggio et al. (24)	Argentina	HF patients (NYHA I–II, LVEF ≤ 40%, QRS ≥ 120 ms)	Lifetime	1 year	+0.94	CRT-P	ID$34,185/QALY	€40,475/QALY	43,680 ID$	Third-party payer	Hybrid decision tree+ Markov model.
							Cost-effective	(PPP conv.)			
Bertoldi et al., (33)	Brazil	NYHA II–IV, public system	20 years	3 years	+1.59	CRT-P	Int$15,723/QALY	€17,420/QALY (PPP conv.)	9,490€–28,470€	Public health system	Hybrid decision-analytic/Markov model.
							Cost-effective				
Almenar et al. (23)	Spain	HF patients (NYHA I–II, LVEF↓, desynchrony, REVERSE cohort)	10 years	2 years	+0.77	CRT-P	€21,500/QALY	€29,762/QALY	32,500€–97,500€	Healthcare payer	Markov decision- analytic model.
							Cost-effective				
Permsuwan et al. (25)	Thailand	HF patients (NYHA III–IV, EF ≤ 35%, QRS prolonged)	Lifetime	2 years	+1.08	CRT-P	THB 104,325/QALY	€3,048/QALY	6,760**€**—20,280**€**	Healthcare payer	Decision tree + Markov model.
							Cost-effective				

**Table 2:** Comparative summary of CRT-D vs. CRT-P across included studies.

**Author**	Country	Population	Time Horizon	Follow Up	QALY (CRT-D)	ICER (CRT-D)	ICER (€ 2024, 3% adj.)	WTP threshold	Perspective	Model type
**Year**										
Feldman et al. (37)	US	HF patients, NYHA III/IV	7 years	2 years	+0.13	$43,000/QALY	€71,446.85/QALY	77,770 €–233,310€	Healthcare payer	Markov simulation model.
						Cost Effective				
Yao et al. (35)	UK	HF patients, LVEF ≤ 35%, NYHA III/IV	7 years	2.5 years	+0.70	€47,909/QALY	€80,925.25/QALY	48,990–146,970	Healthcare payer	Markov model.
						Cost-effective				
Callejo D. et al., (29)	Spain	HF patients (NYHA III–IV, EF ↓, wide QRS	Lifetime	18 months	+0.39	€70 000/QALY;	€105,572.00/QALY	32,500€–97,500€	Public healthcare system	Markov decision-analytic model.
						Not Cost Effective				
Neyt et al. (34)	Belgium	NYHA III/IV, LVEF ≤35%	Lifetime	16 months	+0.55	€57,000/QALY	€83,391/QALY	30,000€-50,000€	Healthcare payer	Markov model.
						Not Cost-effective				
Bertoldi et al. (33)	Brazil	NYHA II–IV	20 years	36 months	+0.57	Int$84,345/QALY	€70,051.01/QALY	9,490€–28,470€	Public health system	Hybrid decision-analytic/Markov model.
						Not Cost-effective				
Gold M. et al., (27)	US	NYHA III/IV		5 years	+1.47	$43,678/QALY	$53,523/QALY	77,770 €-233,310€	Healthcare payer	Proportion-in-state cohort model.
						Cost Effective				
Shah et al., (28)	US	NYHA III, Non-ischemic, QRS 120 ms/<150 ms, LVEF > 20%/ 25%, 65 years, male, LBBB	Lifetime	NR	+0.67	$57,182/QALY	€58,524/QALY	77,770 €- 233,310€	Healthcare payer	Cohort survival model.
						Cost Effective				
Crespo C. et al., (26)	Spain	HF patients, LVEF < 35%, QRS > 120 ms, ischemic/dilated CMP (primary prevention)	Lifetime	5 years	-0.36 (Algorithm 1)	€20,797/QALY	€24,909/QALY	32,500€–97,500€	National health system	Hybrid decision-analytic and Markov model.
					−0.20 (Algorithm 2)	Not Cost Effective				
Hadwiger et al., (30)	Germany	HF patients (NYHA II–IV, EF ≤ 35%, wide QRS)	20 years	2.5 years	+0.57	€24,659/QALY	€26,957.96/QALY	51,600 €	German Statutory Health Insurance (payer)	Cohort Markov model.
						Not cost-effective overall; only for high-risk patients				
Hadwiger et al. (31)	Germany	Real-world claims data (HF)	10 years	6 years	+0.30	€43,643/QALY	€46,286.13/QALY	51,600 €	German Statutory Health Insurance (payer)	Cohort Markov model.
						Not cost-effective overall only in high-risk subgroups				

#### Economic outcomes and interpretation

Cost-effectiveness results were interpreted using standard health economic metrics. A quality-adjusted life year (QALY) combines both quantity and quality of life into a single measure, where 1 QALY represents 1 year lived in perfect health and 0 represents death. The incremental cost-effectiveness ratio (ICER) represents the additional cost required to gain one additional unit of health benefit when comparing two strategies and is calculated as:

*ICER = (Cost intervention—Cost comparator)/(Effect intervention—Effect comparator),*

with effects typically expressed in QALYs.

ICER values were interpreted in relation to willingness-to-pay (WTP) thresholds, defined as the maximum amount a healthcare system or society is willing to pay for one additional QALY gained. An intervention was considered cost-effective when the ICER was below the country-specific WTP threshold applied in the respective study. WTP thresholds were approximated using one times GDP per capita for 2024 obtained from the World Bank database (15).

Cost-effectiveness was interpreted using GDP-based WTP thresholds commonly applied in health economic evaluations. Interventions costing less than one times the gross domestic product (GDP) per capita per QALY gained were considered highly cost-effective, those costing between one- and three-times GDP per capita were considered cost-effective, whereas interventions exceeding three times GDP per capita were considered not cost-effective (16, 17).

#### Cost adjustment

In this review, ICER values were taken as reported in the original studies and preserved in their respective currencies, including euros (€), US dollars ($), British pounds (£), Japanese yen (¥), and Thai baht (THB). To enable comparison across studies and time periods, ICER values were adjusted to 2024, using an annual inflation rate of 3%, a commonly applied standard rate in economic evaluation when harmonizing costs across studies in the absence of uniformly available country-specific healthcare inflation indices. We acknowledge that healthcare inflation differs by country and period; therefore, using a uniform rate may introduce uncertainty in cross-country comparisons, and results should be interpreted primarily within each jurisdiction and against local WTP thresholds (18). To maintain the integrity of the economic evaluations for each country, currency conversions were performed in a separate column in Tables 1, 2. In the rest of the tables, values are presented as originally reported in the respective studies. Although standardized ICER values for 2024 in euros (18) allow cross-country comparison, each ICER was also interpreted against relevant national WTP threshold to assess whether CRT would be considered cost-effective within that country's context. This approach enabled a consistent interpretation of the results within the context of each country.

#### Quality of assessment

In addition, the reporting quality and methodological transparency of each included study were assessed using CHEERS 2022 checklist. Each of 28 items was evaluated as reported, partially reported, or not reported, and an adherence score expressed as a percentage was calculated for every study. Studies were categorized as High (≥85%), Medium (70%–84%), or Low (<70%) reporting quality. Given the nature of model-based economic evaluations, CHEERS adherence was used as an indicator of reporting quality and methodological transparency, which can influence interpretability and potential risk of bias in economic conclusions.

## Results

**Figure 2:**
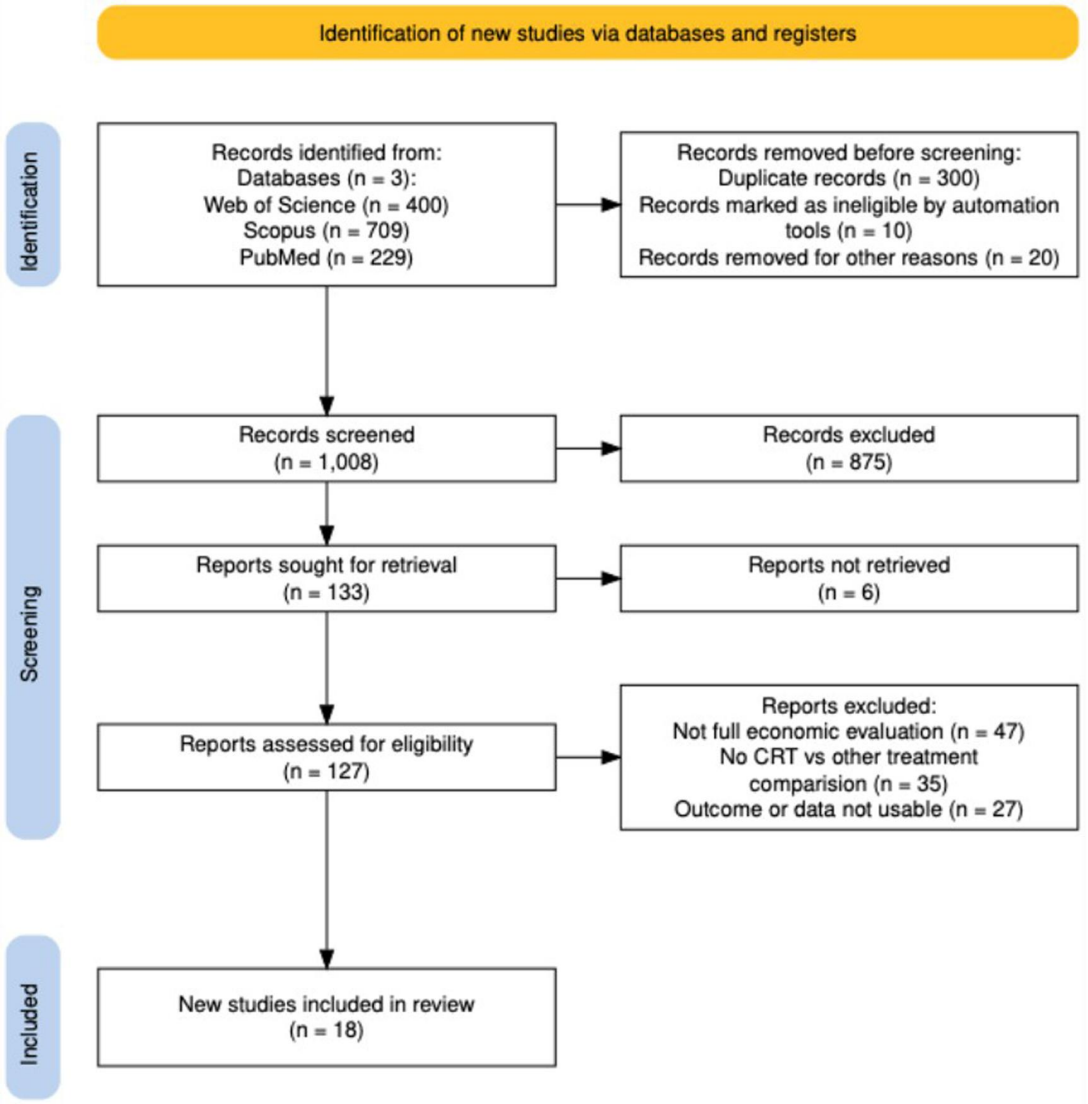
Flow diagram adapted from the PRISMA 2020 Statement. Adapted from “PRISMA 2020 flow diagram template for systematic reviews” by *Page et al.*, licensed under CC BY 4.0.

The selection process and eligibility criteria were structured based on the PRISMA 2020 guidelines (19). A total of 1,338 articles were initially identified. After screening for duplicates, 300 articles were removed. After applying the predefined inclusion and exclusion criteria, 133 full-text articles were assessed for eligibility; 18 studies met the inclusion criteria and were included in the final analysis (Figure 2). Mean patients' age across studies ranged between 60 and 70 years. Among these, 13 articles evaluated CRT plus OMT compared with OMT, and 10 compared CRT-D with CRT-P. Several studies contributed to more than one comparison category. Risk of bias and reporting quality were assessed using the CHEERS 2022 checklist (Supplementary Table S3). Based on the CHEERS 2022 checklist, the quality of the studies ranged from 71% to 96%, with a median quality of 87%. Out of all studies, 17 (94,4%) were rated as high quality (≥85% adherence) (20–36), 1 (5,56%) as medium quality (70%–84%) (37), and none as low quality (<70%). Most studies clearly reported the title, perspective, time horizon, cost and outcome measures, currency year, model structure, and sensitivity analysis. Common gaps were missing pre-registered plans for analysis, limited consideration of subgroup differences in cost-effectiveness, and lack of patient or stakeholder involvement. Overall, the high CHEERS adherence suggests a generally low risk of reporting-related bias, although the identified gaps may affect interpretability in specific domains (e.g., subgroup analyses). The 18 included studies published between 2004 and 2025, spanning Europe (20–23, 26, 29–32, 34–36), North America (27, 28, 37–40), Latin America (24, 33), and Asia (25, 41). Study designs included trial-based cost-effectiveness analyses (21, 27, 32, 35, 37), Markov models (22, 25, 26, 28, 30, 31, 33, 34, 36), decision-analytic frameworks (20, 23, 24, 26, 27, 29, 33), and, less frequently, discrete-event simulations (24, 27, 28, 41). Sensitivity analyses were performed in nearly all studies, mainly using one-way and probabilistic sensitivity analysis (34). ICER estimates were most sensitive to device acquisition and replacement costs, time horizon, survival extrapolation, hospitalization rates, and quality-of-life assumptions. Shorter time horizons may overestimate ICER values for device-based therapies such as CRT because these interventions involve high upfront costs, whereas the clinical benefits, QALY gains, and potential cost offsets from reduced hospitalizations accrue over longer periods. In general, cost-effectiveness improved with longer time horizons, lower device costs, and among patients at higher arrhythmic risk.

### CRT combined with OMT vs. OMT alone

A total of 13 studies evaluated the cost-effectiveness of CRT combined with OMT compared with OMT alone (20–25, 29, 32–37). As summarized in Table 1, economic evaluations consistently demonstrated that CRT, particularly CRT-P, combined with OMT is cost-effective across diverse healthcare settings and countries. Reported incremental QALY gains ranged from +0.43 to +1.98, with ICER values between €3,048–€71,447/QALY gained, remaining mostly within or near accepted willingness-to-pay thresholds. Studies from high-income countries, including the UK (32, 35), the US (42), and Germany (22), reported incremental gains of +0.16 to +1.98 QALYs with ICERs standardized to €12,732–€34,880 in the UK and the US, and €71,447/QALY in Germany. In Belgium, CRT-P yielded +1.31 QALYs with €16,434/QALY (34), while in Brazil, +1.59 QALYs were achieved at €17,420/QALY (33). Evidence from middle- and low-income countries showed even more favorable results. In Thailand, a gain of +1.08 QALYs was reported at only €3,048/QALY (25), compared to +0.94 QALYs with €40,475/QALY in Argentina (24). A multicenter European analysis (32) confirmed these trends, reporting an incremental gain of +0.22 QALYs with an ICER of €33,876/QALY. Taken together, these findings confirm that CRT combined with OMT provides substantial clinical benefit and strong economic value across diverse healthcare systems (23, 36).

### CRT-D vs. CRT-P

In Table 2, most cost-effectiveness evaluations indicate that CRT-D is not cost-effective, with ICER values frequently exceeding country-specific WTP thresholds (26, 28, 29, 31, 33, 34). Across studies, standardized ICER values ranged from approximately €24,909–€105,572/QALY gained (26, 29), with most analyses clustering between €70,005–€83,391/QALY (33–35, 37), exceeding commonly accepted cost-effectiveness thresholds. While a limited number of studies, particularly from the United States, reported ICER values below national WTP thresholds and therefore considered CRT-D cost-effective (27, 28, 37), most analyses conducted in Belgium, Brazil, Germany, and Spain, highlighted limited cost-effectiveness or only marginal value, particularly when the additional benefit of defibrillator therapy was uncertain (26, 30, 31, 33, 34, 37). In other studies CRT-D was found to be more expensive with modest or no additional QALY gains, suggesting that CRT-P may represent a more efficient option for most patients. The cost-effectiveness of CRT-D appears more favorable only in specific high-risk subgroups (30, 31), where the clinical benefit of defibrillator support is more pronounced.

## Discussion

This review assessed the cost-effectiveness of CRT compared with other treatment modalities, including OMT, across high-, middle- and low-income countries. Analysis of 18 studies provided a comprehensive evaluation of CRT strategies and their economic impact on healthcare systems. The results of our systematic review showed that, (1) CRT combined with OMT consistently demonstrates cost-effectiveness compared with OMT, with ICER values generally within the countries accepted WTP thresholds; (2) CRT-P is more cost-effective compared to CRT-D, for most patient populations, while CRT-D may be justified in high-risk subgroups, particularly patients with left bundle branch block (LBBB) and broad QRS. Combining CRT with OMT further improves cost-effectiveness compared with OMT alone, mainly by reducing hospital admissions. This effect is especially relevant in middle- and low-income countries, where healthcare budgets are constrained and efficient use of resources is needed. Across studies, CRT particularly CRT-P has also been associated with lower all-cause and cardiovascular mortality, thereby reinforcing both its clinical and economic value. In comparison with OMT alone, CRT-P combined with OMT consistently demonstrates favorable cost-effectiveness across diverse healthcare settings and patient populations. Across studies, the ICER per QALY gained varied widely, reflecting differences in jurisdiction-specific costs (including device and hospitalization costs), analytic perspective and included cost components, model structure and assumptions (trial-based vs. Markov), time horizon and discounting, and baseline patient risk and event rates. Therefore, ICERs are best interpreted within each country's context and relative to local willingness-to-pay thresholds, rather than compared directly across healthcare systems. Differences in cost-effectiveness across countries are likely driven by variations in willingness-to-pay thresholds rather than intrinsic differences in device performance. When interpreting cost-effectiveness evidence across different healthcare settings, several key aspects should be considered. First, alignment with the local analytic perspective and costing structure (e.g., payer vs. societal perspective) is essential. Second, the time horizon of the analysis should be considered, as shorter horizons may underestimate the long-term benefits of device-based therapies. Third, baseline patient risk particularly arrhythmic risk and assumptions regarding device replacement or complications may substantially influence ICER estimates.

Methodological heterogeneity across included economic models (trial-based analyses, Markov models, decision-analytic frameworks, and discrete-event simulations) likely contributed further to ICER variability, particularly through differences in extrapolation beyond trial follow-up, time horizon, and structural assumptions. Consequently, ICERs should be interpreted in light of model type and assumptions and not compared directly across studies or settings. Three main aspects help in explaining why CRT-P is often preferred over CRT-D: (a) the economic aspect, as CRT-D carries substantially higher upfront costs due to the integrated defibrillator, which can outweigh its incremental benefits in the broader population; (b) the clinical aspect, because the survival advantage of CRT-D over CRT-P tends to be clinically significant only in selected high-risk subgroups, such as those with left bundle branch block (LBBB) and broad QRS; and c) the methodological aspect, differences in study design, modeling assumptions, and patient selection influence cost-effectiveness estimates across settings. Overall, CRT-P is generally more cost-effective than CRT-D, though results vary depending on country and patient subgroup. For CRT-D, economic justification is usually limited to specific high-risk populations, where the added benefits justify the higher cost. In this context, “high-risk” refers to patients with left bundle branch block (LBBB) morphology and markedly prolonged QRS duration (e.g., ≥150 ms), and may also include those with ischemic cardiomyopathy, younger age, and higher baseline arrhythmic risk, in whom the incremental survival benefit of defibrillator capability can justify the higher device cost. The cost-effectiveness of the combination of CRT-P and optimum medical treatment is especially relevant in middle- and low-income countries, where healthcare budgets are constrained and efficient use of resources is critical. Finally, technological and organizational innovations, such as multipoint pacing, cardiac resynchronization therapy–care pathway (CRT-CPW), a structured multidisciplinary management model integrating standardized follow-up and optimization protocols, and wireless stimulation endocardial cardiac resynchronization therapy (WiSE-CRT), a leadless left ventricular endocardial pacing system for patients unsuitable for conventional transvenous CRT, adaptive algorithms, quadripolar leads, and longer battery life, further improve CRT's benefits. By extending device longevity, reducing complications, and enabling personalized therapy, these drivers increase both the clinical benefits and overall cost-effectiveness of CRT.

### Cost-Effectiveness drivers

Several technological and management factors emerged as key drivers of cost-effectiveness in CRT (Table 3). This section focuses on modifiable factors within healthcare systems that may improve the cost-effectiveness of CRT, such as reducing device costs, minimizing complications, and optimizing patient selection and follow-up.

**Table 3:** Cost-Effectiveness drivers.

Drivers	Applied to	Comment
MPP	CRT-P & CRT-D	Multipoint Pacing technology improving pacing sites
CRT-CPW	CRT-P & CRT-D	CRT Care Pathway (CPW), a clinical care model
WiSE CRT	CRT in general	Wireless Stimulation Endocardially (WiSE), alternative CRT
AEE	Mainly CRT-D	Antibiotic-Eluting Envelope to reduce infections
Battery Longevity	Mainly CRT-D	Improved battery life for device durability
AdaptiveCRT	CRT-P & CRT-D	Algorithm that adapts pacing to patient needs
Quadripolar/Bipolar	CRT-P & CRT-D	Types of leads used for improved electrical delivery
rATP	Mainly CRT-D	Reactive Atrial Anti-Tachycardia Pacing function
wICER	CRT-P & CRT-D	Winsorized Incremental Cost-Effectiveness Ratio

Most studies, highlighted multipoint pacing (MPP) (43), adaptive algorithms (44), a structured pathways (CRT-CPW) (45) improved synchronization, optimized patient management, and lowered rehospitalization and follow-up costs, as explanation for favorable or even dominant (cost saving) ICERs. Implementing the CRT-CPW model reduced average hospitalization costs from €19,933 to €17,698, being cost-effective in over 90% of simulations (45). Likewise, MPP and AdaptiveCRT algorithms improved survival and reduced 30-day readmissions, resulting in favorable or even dominant ICERs, demonstrating cost savings and a negative ICER (–€4,137/QALY) over a 6-year horizon (43, 44). Technological improvements such as battery longevity were repeatedly identified as major determinants of long-term cost-effectiveness, as generator replacements substantially increase costs over 5–10 years (45). Extended-life batteries (2.1 Ah) resulted in savings of $15,000 per patient, and system-wide reductions of up to $500 million within six years (39). These findings were confirmed in a six-year model-based study, showing consistent cost savings. Similarly, quadripolar leads reduced re-interventions and hospitalizations, with ICERs as low as £3,692/QALY gained (46). Innovations addressing procedural complications also contributed significantly. The use of antibacterial envelopes during CRT-D replacement reduced infection-related costs, proving to be cost-effective in high-risk subgroups ($50,000–$103,000/QALY) (47). Moreover, WiSE-CRT offered cost-effective solutions for non-responders and patients unsuitable for transvenous implantation, maintaining ICERs below NICE accepted thresholds (39). This technology provided a cost-effective alternative for approximately 30% of non-responders and about 5% of patients unsuitable for transvenous CRT implantation. In addition, the rATP (reactive atrial anti-tachycardia pacing) algorithm has emerged as a promising programming innovation, shown to slow atrial fibrillation progression and reduce stroke and heart failure risk, achieving an ICER of approximately €10,245 per QALY, below Japan's willingness-to-pay threshold (48). Finally, methodological refinements such as the weighted ICER (wICER) improved model precision and identified patient-level predictors of favorable cost-effectiveness, including LBBB morphology and moderately broad QRS (49). Overall, cost-effectiveness drivers confirm that beyond device selection alone, strategic innovations in programming, device design, and patient management are central in maximizing the clinical benefits and economic value of CRT. Emerging physiologic pacing approaches, such as left bundle branch area pacing, may further influence the future economic landscape of CRT by potentially achieving ventricular resynchronization with simpler lead configurations and lower procedural costs.

### Study limitations

Most studies originated from high-income countries, limiting generalizability to middle- and low-income settings. Inclusion of studies from Brazil, Thailand, and Argentina partially addresses this geographic gap in the evaluation of CRT interventions. Additionally, not all studies reported subgroup-specific outcomes (e.g., NYHA class, LBBB, QRS duration), which constrains interpretation and clinical applicability. Furthermore, the literature search was limited to English-language databases, which may have excluded relevant studies published in other languages. Another limitation is that the majority of included studies adopted a healthcare payer or national health-system perspective, potentially overlooking broader societal costs and benefits. Specifically, indirect costs such as productivity losses, return-to-work outcomes, informal caregiving, and caregiver burden were generally not incorporated into the economic models. Exclusion of these societal components may underestimate the full economic value of CRT, particularly in younger or working-age populations. Nonetheless, available subgroup analyses provide useful guidance for evidence-based practice and policy decisions.

## Conclusions

This review confirms that cardiac resynchronization therapy, especially CRT-P, represents a cost-effective treatment for patients with HFrEF who remain symptomatic despite OMT. CRT-D shows variable cost-effectiveness and should be reserved for selected high-risk patients. These findings support prioritizing CRT-P as a high-value therapy within advanced heart failure care.

## Data Availability

The original contributions presented in the study are included in the article/Supplementary Material, further inquiries can be directed to the corresponding author.

## Supplementary material

Supplementary Table S1 PRISMA 2020 Checklist.

Supplementary Table S2 CHEERS 2022 Checklist.

Supplementary Table S3 CHEERS 2022 adherence (reporting quality) of included economic evaluations. The completed PRISMA 2020 and CHEERS 2022 checklists are available as supplementary files accompanying this article.
