# Supplementary material for: Global evidence on the cost-effectiveness of cardiac resynchronization therapy for heart failure: a systematic review
Source: Front Cardiovasc Med. 2026 May 21;13:1766979. doi: 10.3389/fcvm.2026.1766979 (PMC13234864; doi:10.3389/fcvm.2026.1766979)
Supplement: Supplementary Table S1 — PRISMA 2020 Checklist. [file Table1.docx]

| **Section and Topic** | **Item #** | **Checklist item** | **Location where item is reported** |
| --- | --- | --- | --- |
| **TITLE** | | |  |
| Title | 1 | Identify the report as a systematic review. | Title page: “Cost-Effectiveness of Cardiac Resynchronization Therapy for Heart Failure: A Systematic Review.” |
| **ABSTRACT** | | |  |
| Abstract | 2 | See the PRISMA 2020 for Abstracts checklist. | Abstract section, lines 1–12. |
| **INTRODUCTION** | | |  |
| Rationale | 3 | Describe the rationale for the review in the context of existing knowledge. | Introduction, paragraphs 1–3. |
| Objectives | 4 | Provide an explicit statement of the objective(s) or question(s) the review addresses. | Introduction, final paragraph + “Research question and PICOS Framework” in Methods. |
| **METHODS** | | |  |
| Eligibility criteria | 5 | Specify the inclusion and exclusion criteria for the review and how studies were grouped for the syntheses. | “Inclusion and Exclusion Criteria” subsection. |
| Information sources | 6 | Specify all databases, registers, websites, organisations, reference lists and other sources searched or consulted to identify studies. Specify the date when each source was last searched or consulted. | “Search strategy” subsection: PubMed, Scopus, Web of Science, and Cochrane databases searched for studies published from January 2004 to July 2025 using predefined MeSH terms. |
| Search strategy | 7 | Present the full search strategies for all databases, registers and websites, including any filters and limits used. | “Search strategy”, paragraph 2. |
| Selection process | 8 | Specify the methods used to decide whether a study met the inclusion criteria of the review, including how many reviewers screened each record and each report retrieved, whether they worked independently, and if applicable, details of automation tools used in the process. | Not applicable (no risk-of-bias assessment; CHEERS 2022 used instead). |
| Data collection process | 9 | Specify the methods used to collect data from reports, including how many reviewers collected data from each report, whether they worked independently, any processes for obtaining or confirming data from study investigators, and if applicable, details of automation tools used in the process. | Methods → “Data Extraction and Synthesis”: two independent reviewers (K.B., A.B.) extracted data using predefined templates; discrepancies resolved by consensus; no automation tools used. |
| Data items | 10a | List and define all outcomes for which data were sought. Specify whether all results that were compatible with each outcome domain in each study were sought (e.g. for all measures, time points, analyses), and if not, the methods used to decide which results to collect. | Methods → “Data Extraction”: extracted QALYs, ICERs, life-years gained, costs, perspectives, time horizon, model type, country, and cost-effectiveness conclusion. |
|  | 10b | List and define all other variables for which data were sought (e.g. participant and intervention characteristics, funding sources). Describe any assumptions made about any missing or unclear information. | Methods → “Data Extraction and Synthesis”: collected additional data on study design, currency, year, comparator interventions, and cost adjustment; assumptions clarified when information missing. |
| Study risk of bias assessment | 11 | Specify the methods used to assess risk of bias in the included studies, including details of the tool(s) used, how many reviewers assessed each study and whether they worked independently, and if applicable, details of automation tools used in the process. | Not applicable, risk of bias not assessed because studies were economic evaluations; reporting quality assessed via **CHEERS 2022 checklist** (Methods → “Quality of Assessment”). |
| Effect measures | 12 | Specify for each outcome the effect measure(s) (e.g. risk ratio, mean difference) used in the synthesis or presentation of results. | Methods → “Data Extraction”: ICER (€/QALY gained) used as the primary effect measure; QALY differences reported for each comparison. |
| Synthesis methods | 13a | Describe the processes used to decide which studies were eligible for each synthesis (e.g. tabulating the study intervention characteristics and comparing against the planned groups for each synthesis (item #5)). | Methods → “Data Extraction and Synthesis”: grouped by comparison (CRT-P vs OMT; CRT-D vs CRT-P; CRT + OMT). |
|  | 13b | Describe any methods required to prepare the data for presentation or synthesis, such as handling of missing summary statistics, or data conversions. | Methods → “Cost Adjustment”: all ICERs standardized to 2024 euros using PPP and 3% annual discount rate. |
|  | 13c | Describe any methods used to tabulate or visually display results of individual studies and syntheses. | Tables 1,2 (standardized ICERs, QALYs, time horizon, model type); Figures 2–3 (flowchart and summary visuals). |
|  | 13d | Describe any methods used to synthesize results and provide a rationale for the choice(s). If meta-analysis was performed, describe the model(s), method(s) to identify the presence and extent of statistical heterogeneity, and software package(s) used. | Methods → “Data Extraction and Synthesis”: narrative synthesis (no meta-analysis); results grouped by study design and geographic region. |
|  | 13e | Describe any methods used to explore possible causes of heterogeneity among study results (e.g. subgroup analysis, meta-regression). | Results → identified variation by study perspective, country income level, and time horizon; discussed in Discussion (lines 4–8). |
|  | 13f | Describe any sensitivity analyses conducted to assess robustness of the synthesized results. | Results → described sensitivity results from individual studies (e.g., deterministic/probabilistic analyses summarized in each comparison). |
| Reporting bias assessment | 14 | Describe any methods used to assess risk of bias due to missing results in a synthesis (arising from reporting biases). | Not applicable, no statistical synthesis or meta-analysis conducted. |
| Certainty assessment | 15 | Describe any methods used to assess certainty (or confidence) in the body of evidence for an outcome. | Not applicable, not performed; economic evaluation quality judged using CHEERS 2022 adherence levels. |
| **RESULTS** | | |  |
| Study selection | 16a | Describe the results of the search and selection process, from the number of records identified in the search to the number of studies included in the review, ideally using a flow diagram. | Results → Figure 2 (PRISMA flowchart) + text: 1338 identified → 21 included. |
|  | 16b | Cite studies that might appear to meet the inclusion criteria, but which were excluded, and explain why they were excluded. | Results → Figure 2 and text: exclusions for duplication, ineligible comparators, or insufficient economic data. |
| Study characteristics | 17 | Cite each included study and present its characteristics. | Tables 1,2 (country, population, time horizon, model type, ICER). |
| Risk of bias in studies | 18 | Present assessments of risk of bias for each included study. | Not applicable — instead, CHEERS-based reporting quality summarized (Results → “Quality assessment”). |
| Results of individual studies | 19 | For all outcomes, present, for each study: (a) summary statistics for each group (where appropriate) and (b) an effect estimate and its precision (e.g. confidence/credible interval), ideally using structured tables or plots. | Results → Tables 1,2 + narrative summaries in each comparison subsection. |
| Results of syntheses | 20a | For each synthesis, briefly summarise the characteristics and risk of bias among contributing studies. | Results → beginning of each comparison subsection (“As shown in Table X…”). |
|  | 20b | Present results of all statistical syntheses conducted. If meta-analysis was done, present for each the summary estimate and its precision (e.g. confidence/credible interval) and measures of statistical heterogeneity. If comparing groups, describe the direction of the effect. | Not applicable (narrative synthesis only). |
|  | 20c | Present results of all investigations of possible causes of heterogeneity among study results. | Discussion → variation explained by economic perspective, healthcare system, and model design. |
|  | 20d | Present results of all sensitivity analyses conducted to assess the robustness of the synthesized results. | Results → mentioned for studies performing probabilistic or one-way sensitivity analyses. |
| Reporting biases | 21 | Present assessments of risk of bias due to missing results (arising from reporting biases) for each synthesis assessed. | Not applicable (no quantitative synthesis). |
| Certainty of evidence | 22 | Present assessments of certainty (or confidence) in the body of evidence for each outcome assessed. | Results → “Quality assessment”: 94,44% high, 5,56% moderate CHEERS adherence. |
| **DISCUSSION** | | |  |
| Discussion | 23a | Provide a general interpretation of the results in the context of other evidence. | Discussion, paragraphs 1–3. |
|  | 23b | Discuss any limitations of the evidence included in the review. | Discussion → “Study limitations” paragraph. |
|  | 23c | Discuss any limitations of the review processes used. | Discussion → final paragraph (“limited to English-language databases…”). |
|  | 23d | Discuss implications of the results for practice, policy, and future research. | Discussion → last paragraph + “Conclusions”. |
| **OTHER INFORMATION** | | |  |
| Registration and protocol | 24a | Provide registration information for the review, including register name and registration number, or state that the review was not registered. | Methods → PROSPERO ID: CRD420251171292. |
|  | 24b | Indicate where the review protocol can be accessed, or state that a protocol was not prepared. | PROSPERO registration mentioned; no separate published protocol. |
|  | 24c | Describe and explain any amendments to information provided at registration or in the protocol. | Not applicable (no amendments after registration). |
| Support | 25 | Describe sources of financial or non-financial support for the review, and the role of the funders or sponsors in the review. | No specific funding was received. |
| Competing interests | 26 | Declare any competing interests of review authors. | No competing interests.\ |
| Availability of data, code and other materials | 27 | Report which of the following are publicly available and where they can be found: template data collection forms; data extracted from included studies; data used for all analyses; analytic code; any other materials used in the review. | Supplementary materials: PRISMA checklist, CHEERS checklist table, and standardized ICER tables (available upon request). |

*From:*  Page MJ, McKenzie JE, Bossuyt PM, Boutron I, Hoffmann TC, Mulrow CD, et al. The PRISMA 2020 statement: an updated guideline for reporting systematic reviews. BMJ 2021;372:n71. doi: 10.1136/bmj.n71. This work is licensed under CC BY 4.0. To view a copy of this license, visit <https://creativecommons.org/licenses/by/4.0/>
