# Supplementary material for: Global evidence on the cost-effectiveness of cardiac resynchronization therapy for heart failure: a systematic review
Source: Front Cardiovasc Med. 2026 May 21;13:1766979. doi: 10.3389/fcvm.2026.1766979 (PMC13234864; doi:10.3389/fcvm.2026.1766979)
Supplement: Supplementary Table S2 — CHEERS 2022 Checklist. [file Table3.docx]

| **Study** (Author, Year) | **CHEERS adherence** (%) | **Quality category** (High/Medium/Low) |
| --- | --- | --- |
| Maniadakis, 2011 | 91,66% | High |
| Yao et al., 2007 | 90,47% | High |
| Neyt et al., 2013 | 90,00% | High |
| Bertoldi et al., 2013 | 90,00% | High |
| Calvert et al., 2005 | 91,66% | High |
| Feldman et al., 2005 | 84,52% | Medium |
| Hadwiger et al., 2022 | 92.85% | High |
| Hadwiger et al., 2021 | 91,66% | High |
| Callejo et al., 2010 | 94.04% | High |
| Gold et al., 2017 | 94.04% | High |
| Crespo et al., 2020 | 92,85% | High |
| Permsuwan et al., 2020 | 90,47% | High |
| Poggio et al., 2012 | 90,47% | High |
| Almenar et al., 2013 | 91,66% | High |
| Banz et al., 2005 | 91,66% | High |
| Blomstrom et al., 2008 | 90,47% | High |
| Caro et al., 2006 | 90,47% | High |
| Shah et al., 2020 | 91.66% | High |

**Table S3. CHEERS 2022 adherence (reporting quality) of included economic evaluations**

**Footnote:** Quality category defined as **High ≥85%**, **Medium 70–84%**, **Low <70%**.
