# Supplementary material for: Global evidence on the cost-effectiveness of cardiac resynchronization therapy for heart failure: a systematic review
Source: Front Cardiovasc Med. 2026 May 21;13:1766979. doi: 10.3389/fcvm.2026.1766979 (PMC13234864; doi:10.3389/fcvm.2026.1766979)
Supplement: Supplementary Table S3 — CHEERS 2022 adherence (reporting quality) of included economic evaluations. The completed PRISMA 2020 and CHEERS 2022 checklists are available as supplementary files accompanying this article. [file Table4.docx]

| **Country** | **GDP per capita 2024 (USD)** | **GDP per capita (Euro)** | **WTP threshold** |
| --- | --- | --- | --- |
| Belgium |  |  | 30,000-50,000 |
| Brazil | 10,310.5 | 9,490 | 9,490–28,470 |
| Denmark | 71,026.5 | 65,340 | 65,340–196,020 |
| Finland | 53,149.8 | 48,900 | 48,900–146,700 |
| Germany | 56,103.7 | 51,600 | 51,600–154,800 |
| Greece | 24,626.1 | 22,650 | 22,650–67,950 |
| Spain | 35,326.8 | 32,500 | 32,500–97,500 |
| Sweden | 57,117.5 | 52,550 | 52,550–157,650 |
| United Kingdom | 53,246.4 | 48,990 | 48,990–146,970 |
| United States | 84,534.0 | 77,770 | 77,770 – 233,310 |
| Thailand | 7,346.6 | 6,760 | **6,760 – 20,280** |
| Argentina |  |  | 43,680 ID$ |

*Supplementary Table S4. Country-specific willingness-to-pay (WTP) thresholds.* **Note:** WTP thresholds were approximated as one to three times GDP per capita following commonly used WHO cost-effectiveness benchmarks (48,49). WTP thresholds for Argentina and Belgium were taken directly from the respective original studies.
